# Postoperative factors associated with psychological well-being of living kidney donors: results of a retrospective and qualitative study

**DOI:** 10.3389/fpsyg.2024.1377771

**Published:** 2024-07-05

**Authors:** Vasiliki Galani, Viridiana Mazzola, Paco Prada, Guido Bondolfi

**Affiliations:** ^1^Department of Psychiatry, Service of Liaison Psychiatry and Crisis Intervention, Geneva University Hospitals, Geneva, Switzerland; ^2^Department of Psychiatry, Faculty of Medicine, University of Geneva, Geneva, Switzerland

**Keywords:** living kidney donors, kidney transplantation, mental health, postoperative fatigue, donor-recipient relationship, kidney donors’ follow up

## Abstract

**Introduction:**

Kidney transplantation from a living donor is the treatment of choice for end- stage kidney disease. Psychological implications of living kidney donation are of great importance, both during preliminary psychiatric assessment and post-donation follow-up. The identification of risk factors worsening the psychological well-being of living kidney donors (LKDs), before and after donation, remains challenging in terms of research.

**Methods:**

At the University Hospitals of Geneva (HUG), our clinical observations and practice compelled us to establish post-donation follow-ups for LKDs at 6 months and 1 year. Pre-and post-donation sociodemographic, physical, psychological, and psychiatric data was collected from the medical records of 115 LKDs who underwent a complete physical and psychological evaluation during the period 2011-2018. We tested for any potential association between the variables under study.

**Results:**

A qualitative and retrospective analysis of this data highlighted the impact of postoperative factors, such as pain, fatigue, recipient-donor relationship, and fulfillment of donors’ expectations, on the post-donation psychological well-being of LKDs.

**Discussion:**

With regard to these findings, regular post-donation follow-ups, optimal care of postoperative pain and fatigue, as well as a solid therapeutic alliance with LKDs remain key points for clinicians involved in the dynamic process of living kidney donation.

## 1 Introduction

Kidney transplantation from a living donor is the treatment of choice for end-stage kidney disease, and currently represents 40% of all kidney transplantations (Hanson et al., 2019). To ensure the safety of the intervention, a complete physical and psychiatric evaluation of the potential living kidney donor (LKD) must be performed by the transplantation team before the nephrectomy (*primum non nocere*) (Reese et al., 2015).

Post donation, impaired Health-Related Quality of Life (HRQoL), and psychological adjustment disorders have been reported by some authors (Smith et al., 2004). While some physical and psychological parameters are found to have deteriorated during the first 6 months after the procedure, they return to pre-donation levels 1 year post donation and remain above the average observed in the general population (Wirken et al., 2019).

The physical fatigue observed post nephrectomy seems to be progressively resolved during the first 6 months following the donation, but a minority of donors suffers from persistent fatigue for up to 2 years after the procedure (Rodrigue et al., 2020). Postoperative pain also affects the donors’ rehabilitation process and quality of life. Bruintjes et al. (2019) report that the prevalence of chronic post-donation pain is estimated at 5.7% in their cohort.

Post-donation adjustment disorders are also described in literature. Holscher et al. (2018) report a positive post-donation screening for depression at 4.2% for the LKDs in their cohort, and the KDOC study reveals that 16% of donors included in the study presented emotional disturbances post donation (Rodrigue et al., 2018). According to some authors, the prevalence of depression before and after kidney donation remains unchanged (Ong et al., 2021). Manifestations of anxiety are also observed post donation and are possibly related to the recipient’s post-transplantation complications, to changes in the donor-recipient relationship, or to stress factors prior to donation (Rodrigue et al., 2014). Fear of kidney failure has also been described, and post-donation medical follow-ups and reassurance seem to have a positive impact in this regard (Rodrigue et al., 2018; Torres et al., 2021).

Identity, social roles, relationships, and even family dynamics may be found to be altered after kidney donation. Kidney transplantation from a living donor may in some cases strengthen the emotional bonds between the donor and the recipient, reinforce empathy, and improve social interactions within the family. However, disappointment or partially fulfilled expectations on the donor’s part, and feelings of indebtedness and guilt on the recipient’s part, are also described (Ralph et al., 2019). The potential financial burden and a hasty return to work may also have negative impact on the donor’s psychological status (Larson et al., 2019; Lauridsen et al., 2022).

The identification of risk factors worsening the psychological well-being of LKDs, before and after donation, remains a challenge in terms of research. Psychosocial problems, the underlying motivation for the donation, ambivalence, informed consent, and a degree of pessimism regarding the outcome of the transplantation are some preoperative factors that can impact the post-donation psychological well-being of the donor (Dew et al., 2013; Faeder et al., 2015; Timmerman et al., 2016a,b; Kobayashi et al., 2019; Menjivar et al., 2020). Postoperative factors related to the donors’ and recipients’ physical and psychological state after transplantation are also considered to impact the donors’ psychological health post donation (Weizer et al., 1989; Giesing et al., 2004; Timmerman et al., 2016a; Torres et al., 2017; Wirken et al., 2019).

A key point for clinicians involved in preliminary psychiatric assessment is to identify the risk factors likely to bear the most on the psychological well-being of LKDs post donation. Moreover, this understanding is crucial in order to preserve the well-being of LKDs, to organize the medical follow-ups, and to adjust post-donation psychological interventions. With this in mind, and based on clinical observations, we endeavored to identify which physical parameters combine to become risk factors in the short and medium term after kidney donation. We also investigated psychological factors with the potential to impact the psychological well-being of LKDs post donation.

## 2 Patients and methods

### 2.1 Study design and patient population

We conducted a qualitative and retrospective study designed to identify potential sociodemographic, physical, psychological, and psychiatric factors that potentially affect the psychological progress of living kidney donors post donation.

Our database includes 115 living kidney donors who underwent a complete physical and psychological evaluation before kidney donation at the University Hospitals of Geneva during the 2011–2018 period, and who ultimately went on to donate a kidney. We extracted and analysed categorical data recorded during the complete physical and psychological examinations before donation, and during the six-month and one-year post-donation physical and psychological follow-ups.

For the purpose of this study, we extracted from our hospital database the following categorical data concerning the pre-donation period. (i) Sociodemographic data: age, gender, socio-educational level, profession, marital status, religion, nationality. (ii) Data concerning the LKDs’ physical status: experience of pain in the past (diagnosis of chronic pain or documented substantial pain experience during past hospitalisations and/or outpatient consultations), number of past surgical interventions, experience of past anesthesia and healing (presence or absence of related complications during past surgical experiences) and experience of physical fatigue (diagnosis of chronic or recurrent fatigue in the past). (iii) Psychological status: bond with the recipient, reason for the donation, expectations, presence of ambivalence, appreciation of risks relating to the donation. (iv) Psychiatric status and history: personal and familial psychiatric history, history of substance abuse.

We also extracted clinical data concerning the post-donation period. (i) Data about the LKD’s physical status: post-donation complications, pain, fatigue, healing process, length of hospitalization, outcomes for the recipient. (ii) Psychological status: fulfillment of the donor’s expectations, post-donation regret, pain with psychological impact, fatigue with psychological impact, professional and social performance, relationship with the recipient. (iii) Psychiatric status: onset of psychiatric disorders, need for psychotherapy or psychotropic medication post donation, onset or deterioration of substance abuse disorder.

Specifically, for the evaluation of pre-and post-donation pain, we relied on the clinical notes taken by the doctors and nurses who regularly evaluated and compiled the presence of such symptoms. Based on our hospital’s clinical procedures, we classified pain as mild, moderate, or severe according to pain-induced functional and work-related limitations reported by patients and according to the pain treatment required (duration of analgesia prescription). Explicitly, we considered pain as mild if minor annoyance was described by LKDs before or during a limited period after surgery (<2 weeks) and if no interference with everyday life activities and work capacity occurred. We considered the pain as moderate for patients who reported interference of pain with everyday life activities before or after nephrectomy and/or the use of analgesic treatment during the first 3 months after surgery, without interference with work capacity. Finally, we considered the pain as severe if daily activities and work capacity were limited due to pain before or 3 months after surgery and/or if opioid treatment was needed after discharge from the hospital.

Data concerning pre-and post-donation fatigue was also collected from the doctors’ and nurses’ clinical notes before surgery, during the donors’ hospitalization and at follow-ups 6 months and 1 year after the donation. We considered fatigue as the unpleasant sensation of tiredness, a lack of energy, and the need to sleep or rest more. We classified fatigue as mild, moderate, or severe depending on its duration and on how LKDs felt it interfered with their general level of activity or their work capacity. We considered fatigue as mild for LKDs who did not report functional and work limitations due to fatigue before or after nephrectomy or who reported fatigue during a limited period of time (< 2 weeks). Moderate fatigue was noted for LKDs who experienced functional limitations in everyday life due to fatigue before or after surgery, without impact on work capacity, and for whom the duration of fatigue persisted from 2 weeks to 6 months before or after the donation. Fatigue was considered as severe when work capacity and daily activities were impacted for more than 6 months before or after the donation.

Motive, expectations, ambivalence, and appreciation of risks related to the donation were regularly evaluated by the same transplant psychiatrist at HUG during the pre-surgical assessment (anamnestic data). Fulfillment of donors’ expectations, post-donation regret, and recipient-donor relationship were also regularly evaluated by the same transplant psychiatrist during the six-month and one-year follow-ups (anamnestic data).

All this categorical data was collected from the donors’ electronic medical records. Geneva’s Ethics Committee approved the retrospective use of this data for the purpose of this research. In collaboration with the Clinical Research Centre of HUG, we created a platform for the storage of this data (Redcap). The data has been anonymised and its assessment and storage were performed by the principal researcher (VG).

We included all donors who underwent a complete physical and psychological pre-donation assessment at HUG during the 2011–2018 period and who ultimately went on to donate a kidney.

We excluded all donors who underwent a complete physical and psychological pre-donation examination at HUG during the same period, but who were eventually excluded from donation. We also excluded donors who objected to the use of their personal data for the purpose of clinical research.

### 2.2 Analysis

The data we collected consisted of categorical variables. Explicitly, the sociodemographic data was made up of nominal variables, whereas pre-and post-donation data concerning the LKDs’ physical and psychological status was dichotomous and consisted of ordinal variables. Accordingly, and in order to identify risk factors with a potential post-donation impact on the donors’ psychological well-being, we performed a Pearson’s chi-squared test. Specifically, we tested for any potential association between the variables under study, as described below. Frequencies of sociodemographic data were also reported. The statistical analyses were performed with SPSS v.25.

## 3 Results

### 3.1 Study population

Among the 115 LKDs included in this study, 1.7% were 18–25 years old, 27.8% were 25–45 years old, 59.1% were 45–65 years old, and 11.3% were older than 65. Additionally, 66.1% of LKDs were female, 69.6% were married, 73.9% had a secondary education level or higher, and 47.9% were full-time or part-time employed. Overall, 49.6% had already had a history of two to five surgeries, and 81.7 and 80% reported a positive experience from past anesthesia and healing, respectively. Before donation, chronic pain was experienced by 13% of LKDs and recurrent fatigue was reported by 1.7% of LKDs. 45.2% of LKDs donated to siblings and 54.8% donated to genetically unrelated recipients with whom they had a clear and strong emotional bond (partners, friends, close or distant relatives). Among the 115 LKDs, 27.8% wanted to improve the recipient’s quality of life, 8.7% wanted to improve their couple’s or family’s quality of life, and 32.1% presented combined motivations. 91.3% showed no ambivalence before donation and 87.8% had an optimal appreciation of risks related to the donation during the preliminary assessment. In our cohort, 32.2% had a psychiatric history and 8.7% were known for substance disorders.

Data frequencies of the study cohort are shown in Table 1.

**Table 1:** Data frequencies of the study cohort.

Age	18–25	2 (1.7%)
	25–45	32 (27.8%)
	45–65	68 (59.1%)
	>65	13 (11.3%)
Gender	Female	76 (66.1%)
	Male	39 (33.9%)
Marital status	Married	80 (69.6%)
	Divorced	12 (10.4%)
	Single	9 (7.8%)
	Widower	7 (6.1%)
	Other	7 (6.1%)
Educational level	Secondary vocational education	53 (46.1%)
	Higher studies	32 (27.8%)
Profession	Full-time employed	34 (29.6%)
	Part-time employed	21 (18.3%)
	Independent	18 (15.7%)
Ambivalence	Residual	1 (0.9%)
	Absent	105 (91.3%)
	Not known	9 (7.8%)
Risk appreciation	Optimal	101 (87.8%)
	Partial	6 (5.2%)
Past psychiatric history	Yes	37 (32.2%)
	No	67 (58.3%)
	Not reported	11 (9.6%)
Past substance abuse history	Yes	10 (8.7%)
	No	103 (89.6%)
	Not reported	2 (1.7%)

### 3.2 Post kidney donation data

34 (29.6%) of the 115 LKDs included in this study were screened positive for emotional difficulties or psychiatric disorders post donation. These complications were mostly (13%) screened during the first 2 weeks post donation, 3.5% between the third week and the third month post donation, 7% between the third and the sixth month post donation, and 6.1% after the sixth month post donation. Five donors (4.5%) were screened positive for depression and one donor (0.9%) suffered a psychotic episode before their discharge from hospital. 14 (12.2%) donors received psychotropic medication post donation. 47 (40.9%) donors described the recipient-donor relationship post donation as unchanged, 11 (9.6%) reported it as improved, and 5 (4.3%) reported it as deteriorated. One donor (0.9%) had regrets about the donation, and 60% deemed their expectations related to the donation to be fulfilled.

### 3.3 Significant associations between psychological well-being post donation and other variables under study

The analysis of our data showed that some of the studied variables were statistically associated with adverse psychological effects post donation. Indeed, post-donation fatigue was associated with psychological health post donation (Chi^2^ = 16.734, df = 6, *p* = 0.010). Moreover, among the 34 donors who experienced emotional difficulties or psychiatric disorders after the donation, 18 (52.9%) also experienced post-donation fatigue. This percentage is greater than the prevalence of post-donation fatigue among our total cohort (29.6%). Six donors (17.6%) reported persistent fatigue for up to three to 6 months post donation. Post-donation fatigue was also statistically associated with the need for post-donation psychological support (Chi^2^ = 29.179, df = 6, *p* < 0.001) and with post-donation prescription of psychotropic treatment (Chi^2^ = 19.994, df = 6, *p* = 0.003).

While physical complications were not associated with the donors’ post-donation psychological well-being, statistically significant associations were found between adverse psychological effects and post-donation pain with a psychological impact (Chi^2^ = 58.525, df = 4, *p* = 0.000), and with fatigue with a psychological impact (Chi^2^ = 54.670, df = 4, *p* < 0.001). Pain with a psychological impact post donation was also associated with prolonged hospitalization (Chi^2^ = 9.656, df = 4, *p* = 0.047).

In addition, the data analysis highlighted a statistically significant association between the donors’ psychological progress post donation and the partial fulfillment of the donors’ expectations post donation (Chi^2^ = 42.881, df = 6, *p* < 0.001). Partial fulfillment of expectations is also associated with the need for psychological support post donation (Chi^2^ = 42.980, df = 6, *p* < 0.001) and with recipient-donor relationship progression post donation (Chi^2^ = 93,259, df = 9, *p* < 0,001). Among the seven donors who deemed their expectations to be partially fulfilled, three donors described their relationship to the recipient as deteriorated.

The donors’ psychological progress post donation is also associated with the recipients’ health status after the kidney transplantation (Chi^2^ = 28.881, df = 6, *p* < 0.001). For the 34 donors who experienced emotional difficulties or psychiatric disorders post donation, only one recipient faced post-transplantation complications.

Professional performance and psychological well-being post donation are also associated in a statistically significant manner (Chi^2^ = 9.593, df = 4, *p* = 0.48). Professional performance remained unchanged for the majority of LKDs of our cohort (92.2%).

There were no statistically significant associations between donors’ psychological health post donation and the sociodemographic variables under study. Neither were any statistically significant associations found between preoperative psychological and psychiatric variables (motive for donation, risk appreciation, ambivalence, psychiatric history) and psychological progress post donation.

**Figure 1:**
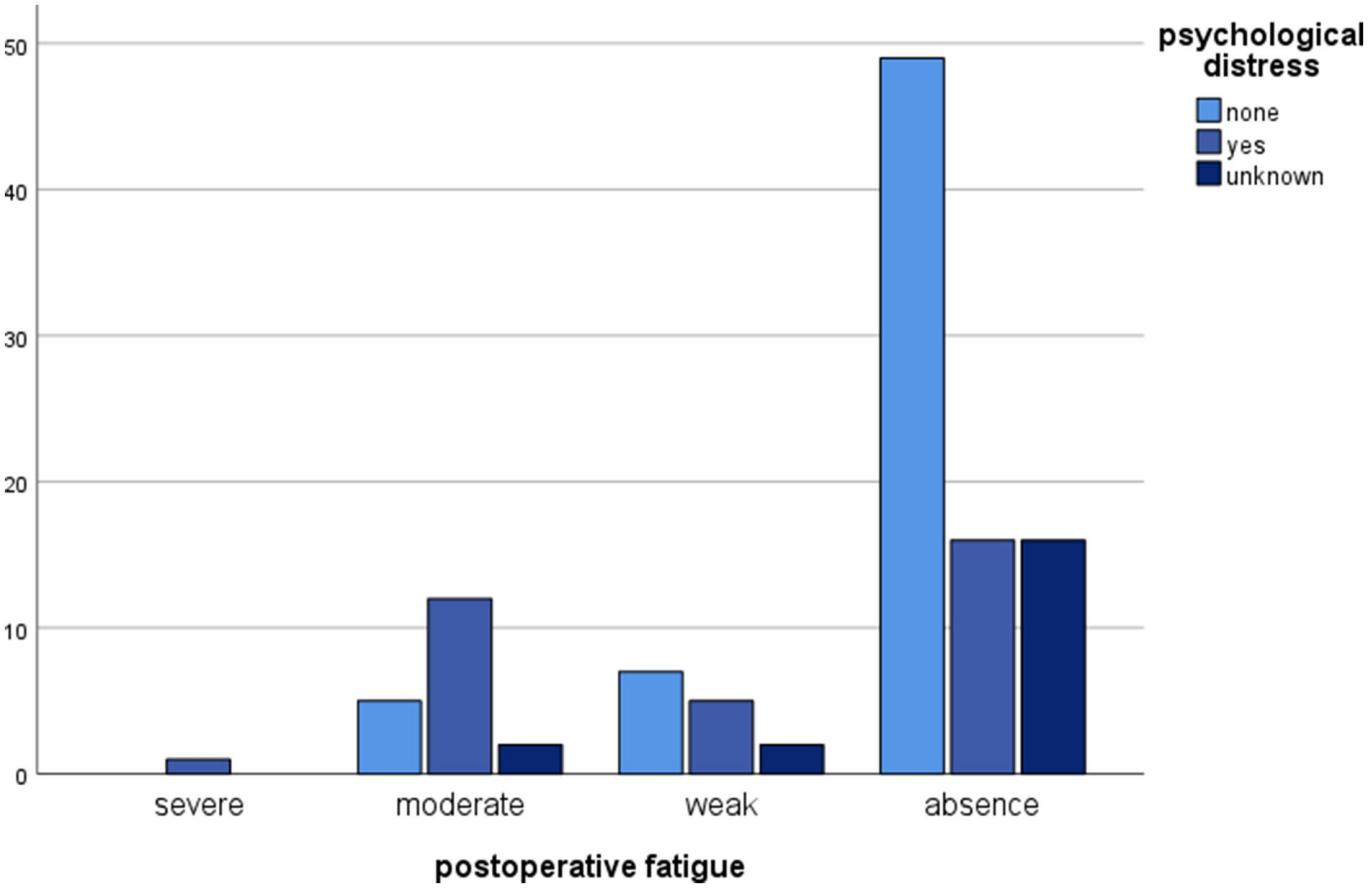
Clustered bar chart of postoperative fatigue by psychological distress categories demonstrating their association. The height of each bar represents the total number of observations.

**Figure 2:**
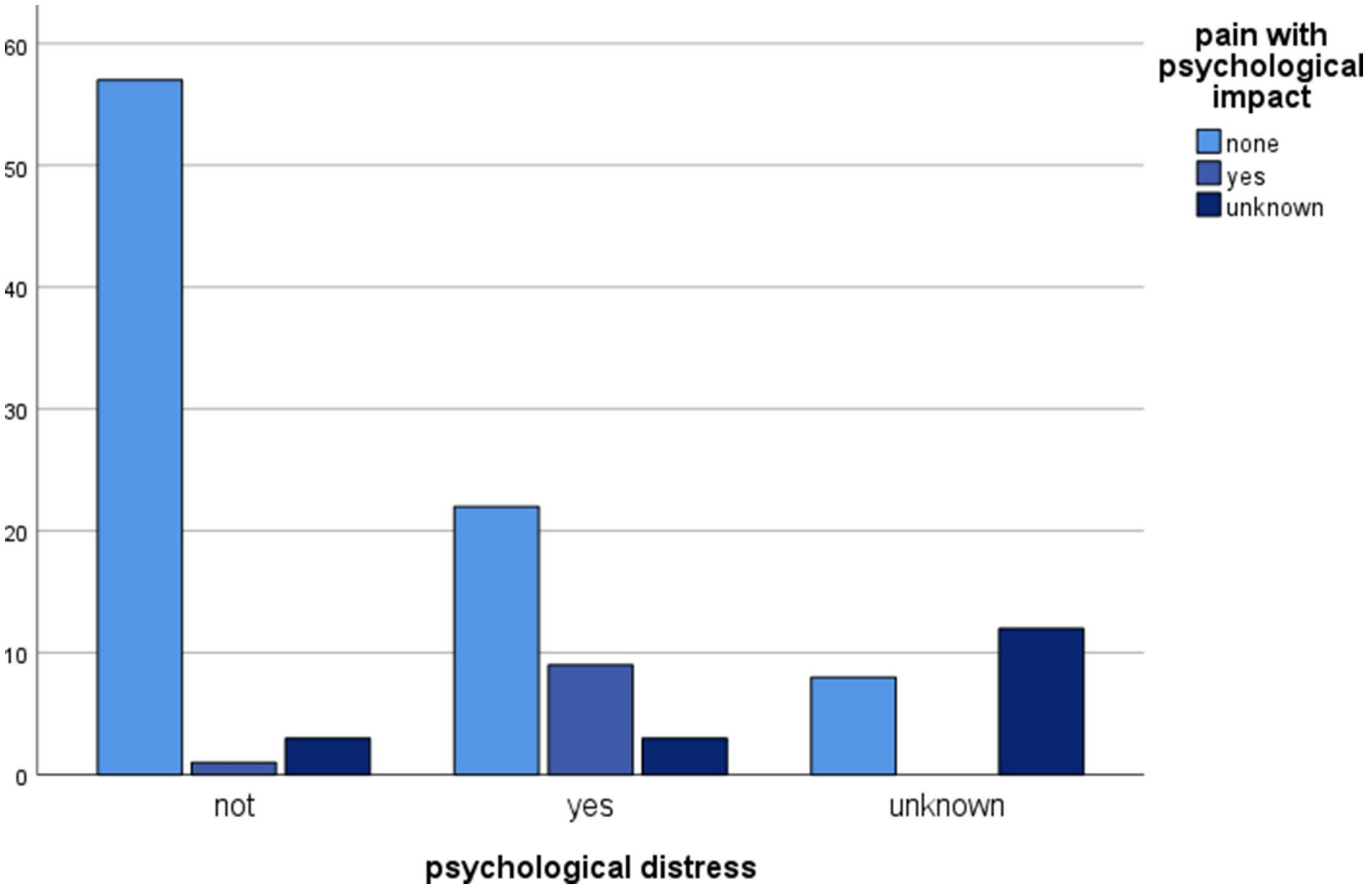
Clustered bar chart of psychological distress by pain with psychological impact categories showing a significant impact of pain on mental status post donation. The height of each bar represents the total number of observations.

**Figure 3:**
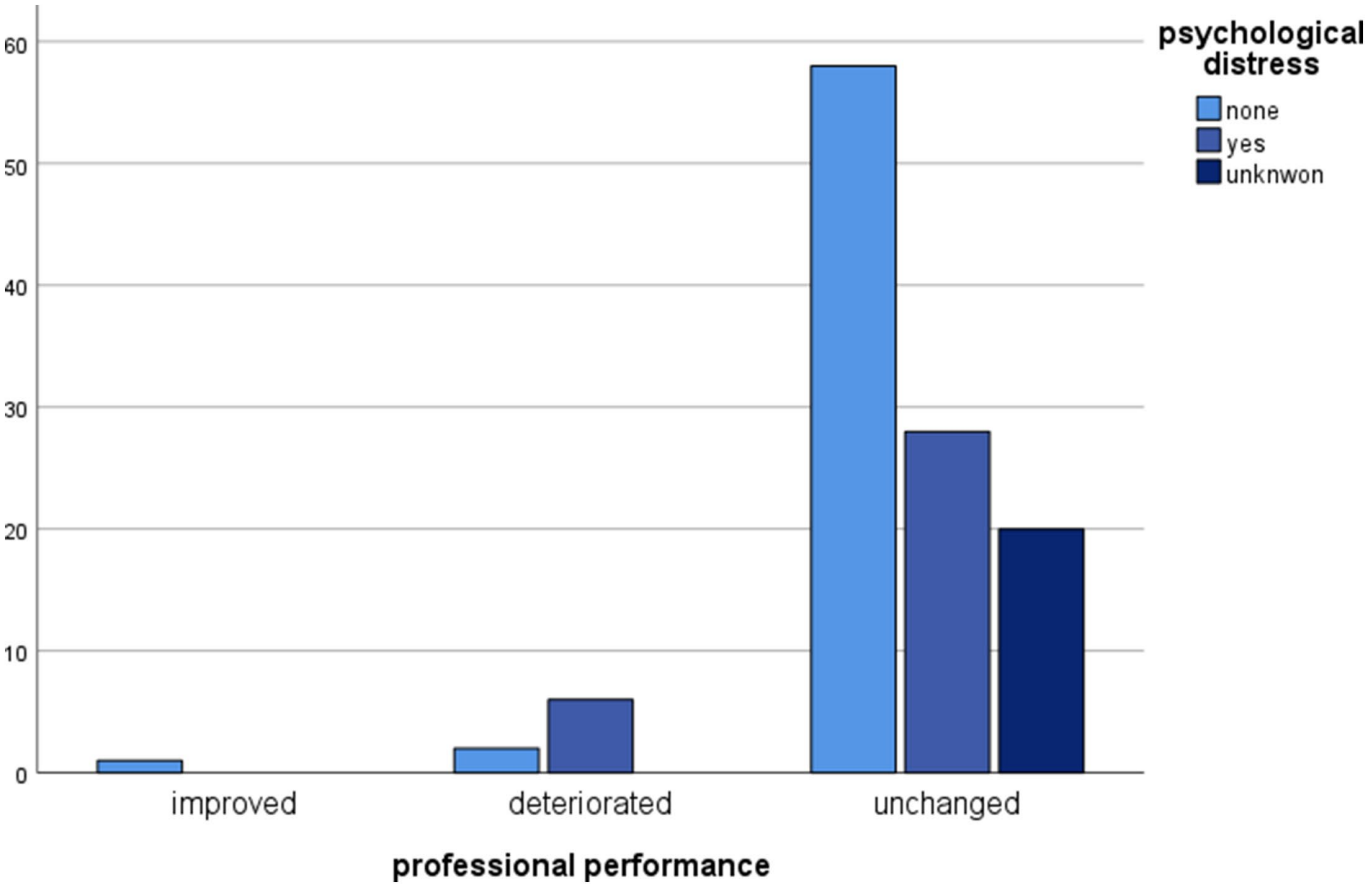
Clustered bar chart of professional performances by psychological distress categories showing an unchanged professional performance for the majority of LKDs. The height of each bar represents the total number of observations.

**Figure 4:**
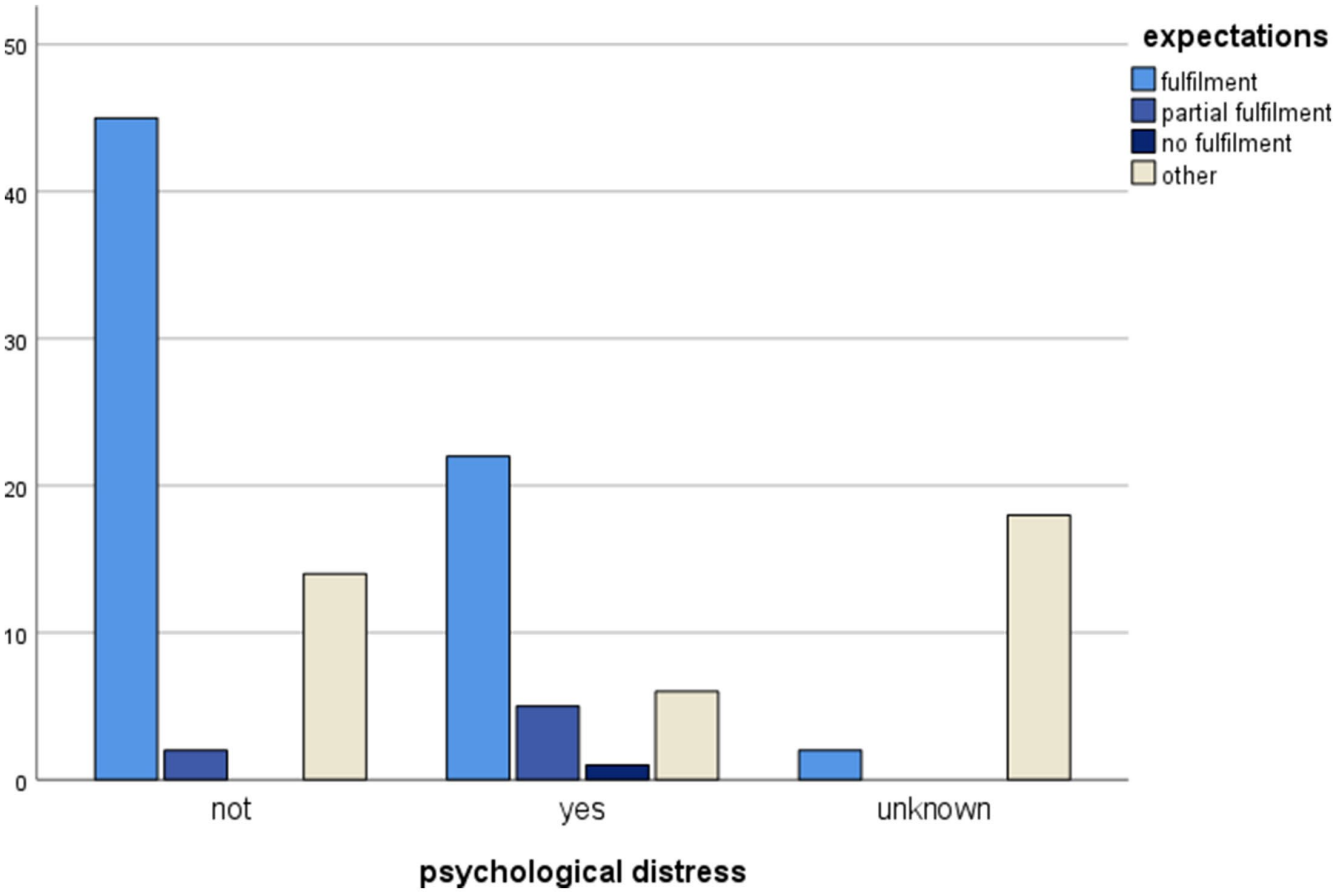
Clustered bar chart of psychological distress by expectations categories illustrating the association of psychological distress with partial fulfilled expectations. The height of each bar represents the total number of observations.

Statistically significant associations between psychological progress post donation and other variables are shown in Figures 1–4.

## 4 Discussion

In this study, we included 115 subjects who had donated a kidney to a sibling or an emotionally related recipient between 2011 and 2018 at the HUG. The primary objective of our study is to identify potential risk factors with an impact on the donors’ post-donation psychological health. Living kidney donation can potentially affect several domains of LKDs’ life in the short, medium and long term: physical health, postoperative rehabilitation process with appearance or accentuation of pain and fatigue, LKDs’ expectations and their relationship with the recipient, work capacity and financial status. In such a context, a state of vulnerability prior to donation may constitute a risk factor for LKDs’ health post donation. Addressing questions about the role of the prior medical history on LKDs’ progress post donation remains also important. Here we are going to discuss the results of our study and their implication on the clinical practice while taking care of LKDs before and after donation.

Our data analysis argues in favor of the absence of any significant correlation between the donors’ psychiatric or substance abuse history and their psychological state post donation. Although these findings are aligned with some studies, other authors support that the psychiatric history and the psychological well-being of donors post donation are significantly associated (Timmerman et al., 2016a,b; Fox et al., 2021). The role of the psychiatric assessment prior to donation is crucial in order to ensure the safety of the donors and protect them from psychological issues post donation. Content and process of psychosocial screening vary between transplantation centers and countries (Massey et al., 2018). Indicators of depression, suicidal tendencies, and low coping abilities were considered as rejection criteria by clinicians in a recent study (Ashkenazi et al., 2023). Despite the diversity of data concerning the role of prior psychiatric history on LKDs’ progress post-donation, it is generally recommended to perform close medical follow-ups for donors who have suffered from mental or substance abuse disorders in the past.

Sociodemographic data before donation were not found to be associated with post-donation psychological health in our study. Psychological variables before donation were neither associated with mental health after living kidney donation. The thorough evaluation of the reasons for donation, donors’ expectations, ambivalence and risk appreciation during the preliminary psychosocial assessment in order to validate LKDs’ eligibility could explain such results. In clinical practice, the presence of a residual ambivalence related to the decision to donate, selective comprehension of risks and the presence of unrealistic expectations necessitate further attention and some authors propose targeted interventions in order to assist potential LKDs in their decisional process (Rodrigue et al., 2006; Dew et al., 2013).

The analysis of our data shows that physical variables before donation were not associated with the post-donation psychological status of LKDs. We could formulate the hypothesis that subjects with severe physical comorbidities were excluded from donation during the preliminary medical evaluation and LKDs selected had a health status above average.

However, post-donation physical outcomes did have a psychological impact on the population under study. In our cohort, post- donation fatigue was found to be associated with LKDs’ mental health after donation. In this study, donors suffering from fatigue after nephrectomy were also more likely to receive psychological support and be prescribed psychotropic treatment during the recovery period. Indeed, post-donation fatigue in LKDs was more frequently observed during the first 2 weeks following the procedure (14.8% of the 115 LKDs included in the study, 29.4% among donors with adverse psychological effects post donation), while six donors reported persistent fatigue between the third and the sixth month post donation. This might be explained by the fact that post-donation fatigue precipitates the onset or accentuation of emotional sensitivity (sadness, anxiety, complaining). Or it could be that emotional discomfort amplifies the feeling of fatigue post donation. For some authors, fatigue has both physical and psychological components that should be taken into account when caring for LKDs and surgical patients (Aadahl et al., 2002; Rodrigue et al., 2020; Oudmaijer et al., 2022).

Pain with a psychological impact post donation was also significantly associated with post-donation psychological well-being of LKDs, the prescription of psychotropic treatments and the duration of hospitalization. These findings shine a light on the role of pain during the post-donation recovery period. Psychological interventions targeting peri-operative stress, anxiety, negative anticipations, and depressive symptoms may be conducive to the optimal postoperative recovery of donors and surgical patients (Gorsky et al., 2021). Further research in this domain could contribute to the optimisation of LKDs’ care post donation (Katvan et al., 2022).

Partial fulfillment of donors’ expectations had an impact on the psychological well-being of LKDs post donation according to the results of this study. In our cohort, seven donors had considered their expectations to be partially fulfilled, and three among them assessed their relationship to the recipient to have deteriorated. They reported feelings of disappointment and unfairness and considered their lifestyle to be deteriorated post donation. These adverse effects were related to donor-recipient relationship and care received post donation, even in the case of the recipient’s favorable progress post transplantation. Our findings seem to be supported by existing literature and emphasize the fact that kidney donation remains a dynamic process that continues months after the transplantation (Clemens et al., 2006; Pistorio et al., 2019). Donors’ expectations about kidney donation are rigorously examined during the preliminary psychosocial assessment, as the partial or complete fulfillment of such expectations has an impact on the psychological well-being of LKDs post donation (Medved et al., 2019). Working on donors’ expectations before the donation and close medical follow-up post donation are areas of interest and should help optimize the patients’ care (Rodrigue et al., 2006; Dew et al., 2013).

According to our analysis, professional performance and psychological health post donation were also found to be associated. Professional performance remained unchanged for the majority of LKDs (92.2%) included in our study. The anticipated return to work after donation and the financial burden related to the donation have already been described in the available literature. Their impact on the donors’ recovery and well-being is a crucial point and must be taken into account during post-nephrectomy care (Fehrman-Ekholm and Tydèn, 2004; Wirken et al., 2019). Close medical follow-ups for donors who experienced prolonged hospital stay and donation-related complications, as well as the identification of pre-donation factors associated with perceived financial burden post-donation are challenging points in the care of LKDs (Ruck et al., 2018).

## 5 Conclusion

This study confirms that the majority of LKDs have a generally positive psychological experience post donation. Postoperative fatigue and pain, changes to the donor-recipient relationship dynamics, and professional performance after donation were found to be associated in a significant manner with the post-donation psychological well-being of LKDs. These findings underline the fact that kidney donation is a dynamic process with an impact on psychological well-being and quality of life during the post donation period. Regular post-donation follow-ups, treatment of postoperative fatigue and pain, as well as the establishment of a solid therapeutical alliance with LKDs can contribute to the optimisation of the care setting for LKDs.

Our study has several limitations. Firstly, the retrospective nature of our study limited the availability of the data derived from the medical records of the LKDs included in our cohort. The lacking information consists mainly of the physical parameters before donation and of post-donation data from LKDs who live abroad and whose medical follow-up is conducted in medical centers located in other countries. Another limitation due to the retrospective nature of our study was that we were not able to employ specific scales and questionnaires. Rating pain and fatigue remains a challenging point in research and in the case of our study we had only anamnestic data from clinicians, but not donors’ self-assessed questionnaires. The present study underscores the importance of using appropriate scales and questionnaires in our clinical practice. Yet another limitation during the extraction of the data concerning the recipients’ post-transplantation progress was the fact that their medical records were not accessible; all the relevant information was collected from the LKDs’ post-donation medical records. This missing data was taken into account during the analysis in order to minimize its impact on our results. In addition, the preliminary psychiatric assessment may differ between transplantation centers and this can potentially impact the results concerning physical and psychological outcomes post donation. Despite these limitations, our study indicates the need to create standardized protocols during preliminary assessments and post-donation follow-ups and to consider variables such as fatigue, pain, donor-recipient relationship, and professional performance.

## Data availability statement

The data analyzed in this study is subject to the following licenses/restrictions: access limited to the main investigator of this study. Requests to access these datasets should be directed to vasiliki.galani@hcuge.ch.

## Ethics statement

The studies involving humans were approved by Commission cantonale d’éthique de la recherche CCER. The studies were conducted in accordance with the local legislation and institutional requirements. The ethics committee/institutional review board waived the requirement of written informed consent for participation from the participants or the participants’ legal guardians/next of kin because this study being retrospective, it was difficult to obtain informed consent from many donors living abroad or in other cantons. This study aims to contribute to the improvement of living kidney donors’ care in the future.

## Author contributions

VG: Writing – original draft, Writing – review & editing. VM: Writing – review & editing. PP: Writing – review & editing. GB: Writing – review & editing.
